# Supplementary material for: Genetic Biomarkers Associated with Dynamic Transitions of Human Papillomavirus (HPV) Infection–Precancerous–Cancer of Cervix for Navigating Precision Prevention
Source: Int J Mol Sci. 2025 Jun 23;26(13):6016. doi: 10.3390/ijms26136016 (PMC12250414; doi:10.3390/ijms26136016)

**Supplementary Table S1. Results of internal and external validation of the dynamic HPV infection–Precancerous–Cancer of Cervix model.**

| | **Internal validation** | **External validation** |
|---|---|---|
| **Information source** | Chen et al., 2011 [3]<br>Koong et al., 2006 [2] | IOC/IARC 2023 [44]<br>Palmar et al., 2024 [45] |
| **Incidence of cervical cancer** | 26.3 | 19.9 |
| **Expected number of cervical cancers** | 2364 | 1787 |
| **Prevalence of HPV infection (%)** | 12.7 | 10.3 |
| **Inter-screening interval (year)** | 3 | 2 |
| **Attendance rate (%)** | 52.2 | 43.7 |
| **Number of cervical cancers in simulated cohort** | 2386 | 1793 |
| $\chi^2$ **value** | 0.21 | 0.02 |
| ***p*-value** | 0.65 | 0.88 |

**Supplementary Table S2. Estimated risk of developing cervical cancer by risk score percentile.**

| Percentile | Lifetime Risk (%) | RR |
|---|---|---|
| 10 | 0.113 | 0.32 |
| 20 | 0.216 | 0.42 |
| 30 | 0.237 | 0.56 |
| 40 | 0.533 | 0.74 |
| 50 | 0.751 | 1.00 |
| 60 | 0.767 | 1.30 |
| 70 | 1.078 | 1.86 |
| 80 | 1.685 | 3.04 |
| 90 | 2.488 | 9.53 |

**Supplementary Table S3. Simulated relative risk of cervical cancer by precision prevention strategies with 5.1% prevalence of HPV infection.**

| | Relative Risk (95% CI) | | | | | | | | | |
|---|---|---|---|---|---|---|---|---|---|---|
| **Risk Score Percentile** | **<20%** | | **20-40%** | | **40-60%** | | **60-80%** | | **>80%** | |
| **Prevention Strategy** | **HPV testing** | | **HPV testing** | | **HPV testing** | | **HPV testing** | | **HPV testing** | |
| | - | + | - | + | - | + | - | + | - | + |
| **Pap Smear Screening by Inter-screening Interval** | | | | | | | | | | |
| **1 yr** | 0.75 (0.26,2.16) | 0.53 (0.39,0.73) | 0.58 (0.46,0.73) | 0.49 (0.31,0.78) | 0.58 (0.46,0.74) | 0.49 (0.42,0.57) | 0.51 (0.46,0.58) | 0.48 (0.41, 0.57) | 0.47 (0.41, 0.54) | 0.45 (0.41, 0.50) |
| **3 yr** | 0.88 (0.32, 2.41) | 0.49 (0.36, 0.68) | 0.55 (0.44, 0.70) | 0.53 (0.34, 0.83) | 0.67 (0.53, 0.85) | 0.56 (0.48, 0.65) | 0.57 (0.49, 0.67) | 0.50 (0.45, 0.57) | 0.51 (0.44, 0.58) | 0.48 (0.43, 0.53) |
| **5 yr** | 1.00 (0.38,2.66) | 0.63 (0.47,0.84) | 0.62 (0.40, .95) | 0.57 (0.45,0.72) | 0.71 (0.57,0.89) | 0.63 (0.54,0.73) | 0.59 (0.53,0.67) | 0.54 (0.46,0.64) | 0.59 (0.51,0.67) | 0.52 (0.47,0.57) |
| **HPV Vaccination + Pap Smear Screening by Inter-screening Interval** | | | | | | | | | | |
| **1 yr** | 0.50 (0.15, 1.66) | 0.33 (0.23, 0.46) | 0.31 (0.23, 0.42) | 0.31 (0.18, 0.53) | 0.25 (0.20, 0.31) | 0.27 (0.20, 0.37) | 0.35 (0.30, 0.40) | 0.30 (0.24,0.36) | 0.37 (0.33, 0.41) | 0.35 (0.30, 0.41) |
| **3 yr** | 0.50 (0.15,1.66) | 0.38 (0.23,0.46) | 0.53 (0.42,0.68) | 0.42 (0.26,0.68) | 0.47 (0.40,0.55) | 0.43 (0.33,0.56) | 0.46 (0.41,0.52) | 0.44 (0.37,0.52) | 0.56 (0.51,0.62) | 0.55 (0.48,0.63) |
| **5 yr** | 0.88 (0.32,2.41) | 0.63 (0.28,0.54) | 0.57 (0.45,0.72) | 0.49 (0.31,0.78) | 0.63 (0.54,0.73) | 0.53 (0.41,0.68) | 0.59 (0.53,0.67) | 0.50 (0.43,0.59) | 0.69 (0.61,0.78) | 0.52 (0.32,0.41) |
| **HPV Vaccination** | | | | | | | | | | |
| | 0.97 (0.75,1.25) | 1.00 (0.38,2.66) | 1.04 (0.85,1.27) | 1.02 (0.70,1.48) | 0.94 (0.82,1.07) | 1.04 (0.85,1.28) | 0.97 (0.87,1.07) | 0.96 (0.84,1.09) | 0.94 (0.87,1.03) | 0.94 (0.84,1.05) |

**Supplementary Figure S1. Distribution of overall risk score and the risk score of HPV-positive and HPV-negative women.**

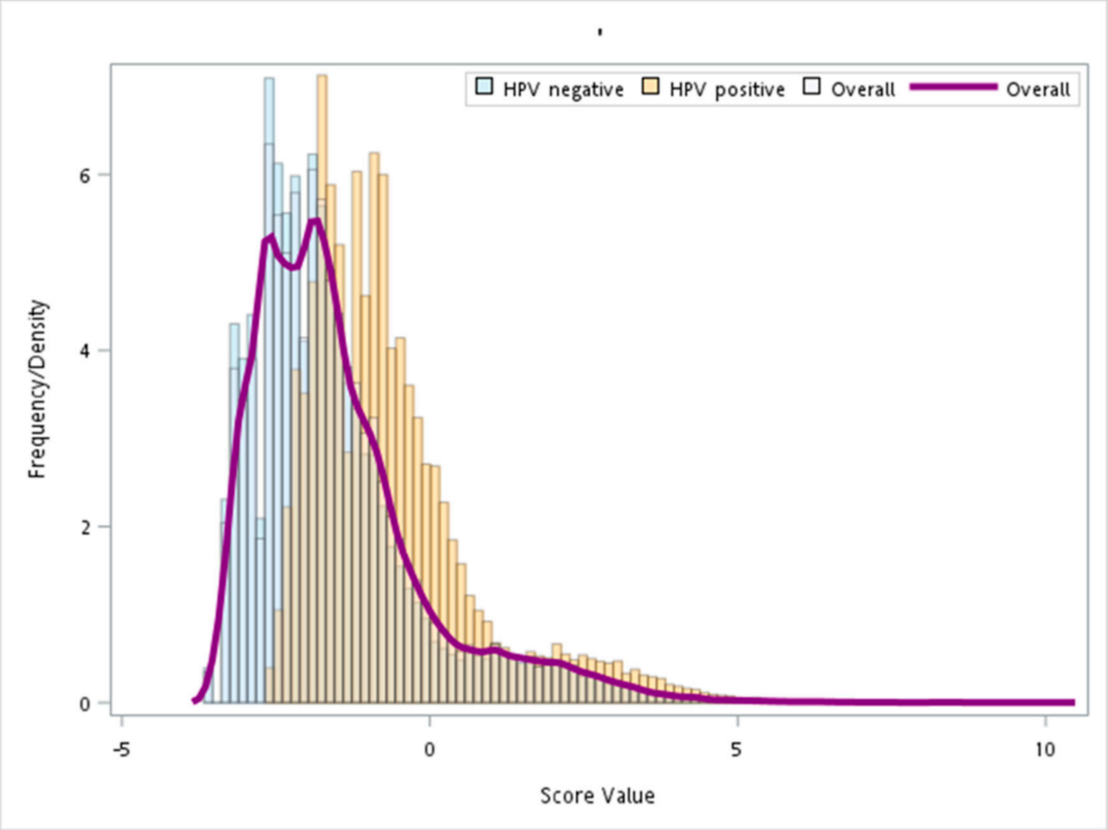

**Supplementary Figure S2. Distribution of overall risk score and the risk score of HPV-positive (a) and HPV-negative (b) women along with the risk sores of six illustrative cases.**

**(a) Women with positive HPV infection (Case A, Case B, and Case C).**

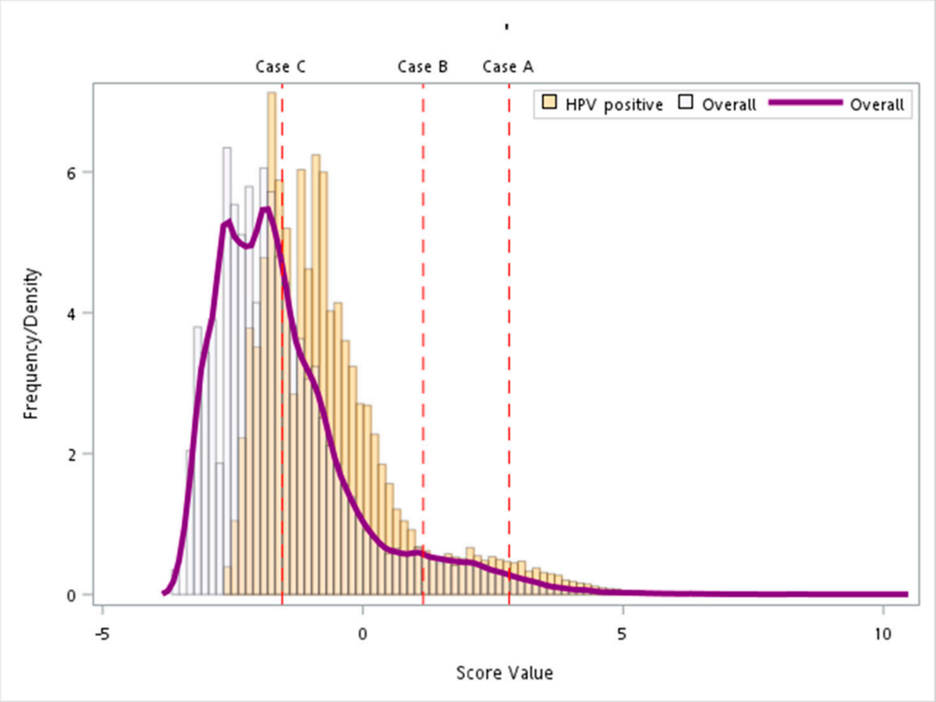


**(b) Women with positive HPV infection (Case D, Case E, and Case F).**

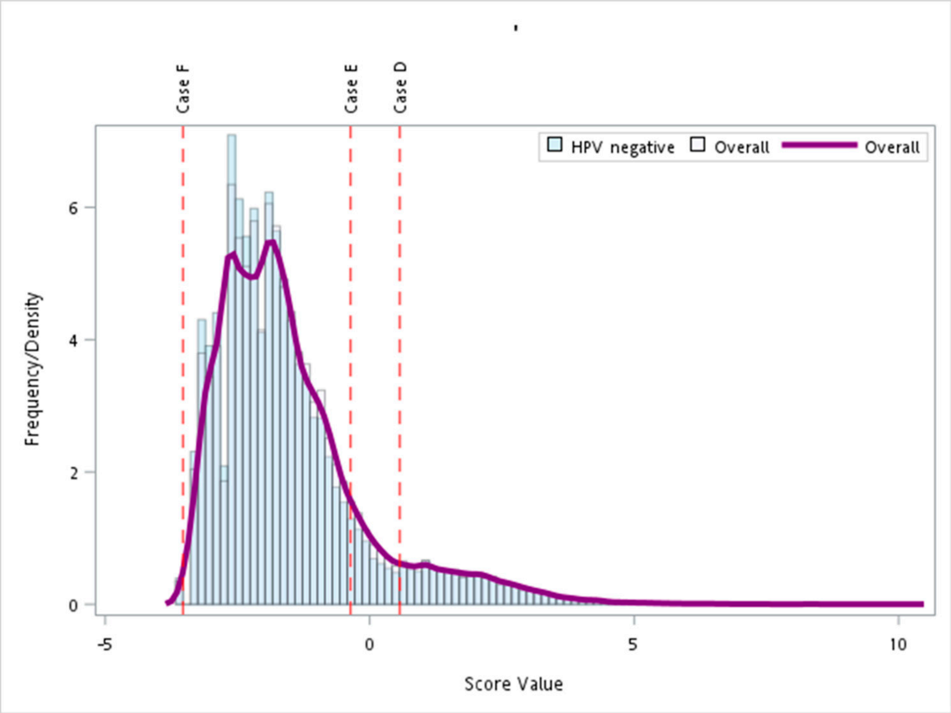

Supplement: Supplementary file 1 [file ijms-26-06016-s001.zip › ijms-3598802-supplementary.pdf]
